# Correction: Force field generalization and the internal representation of motor learning

**DOI:** 10.1371/journal.pone.0227963

**Published:** 2020-01-16

**Authors:** Alireza Rezazadeh, Max Berniker

The images for Figs 1 and 2 are incorrectly switched. The image that appears as Fig 1 should be Fig 2, and the image that appears as Fig 2 should be Fig 1. The figure captions appear in the correct order.

**Fig 1:**
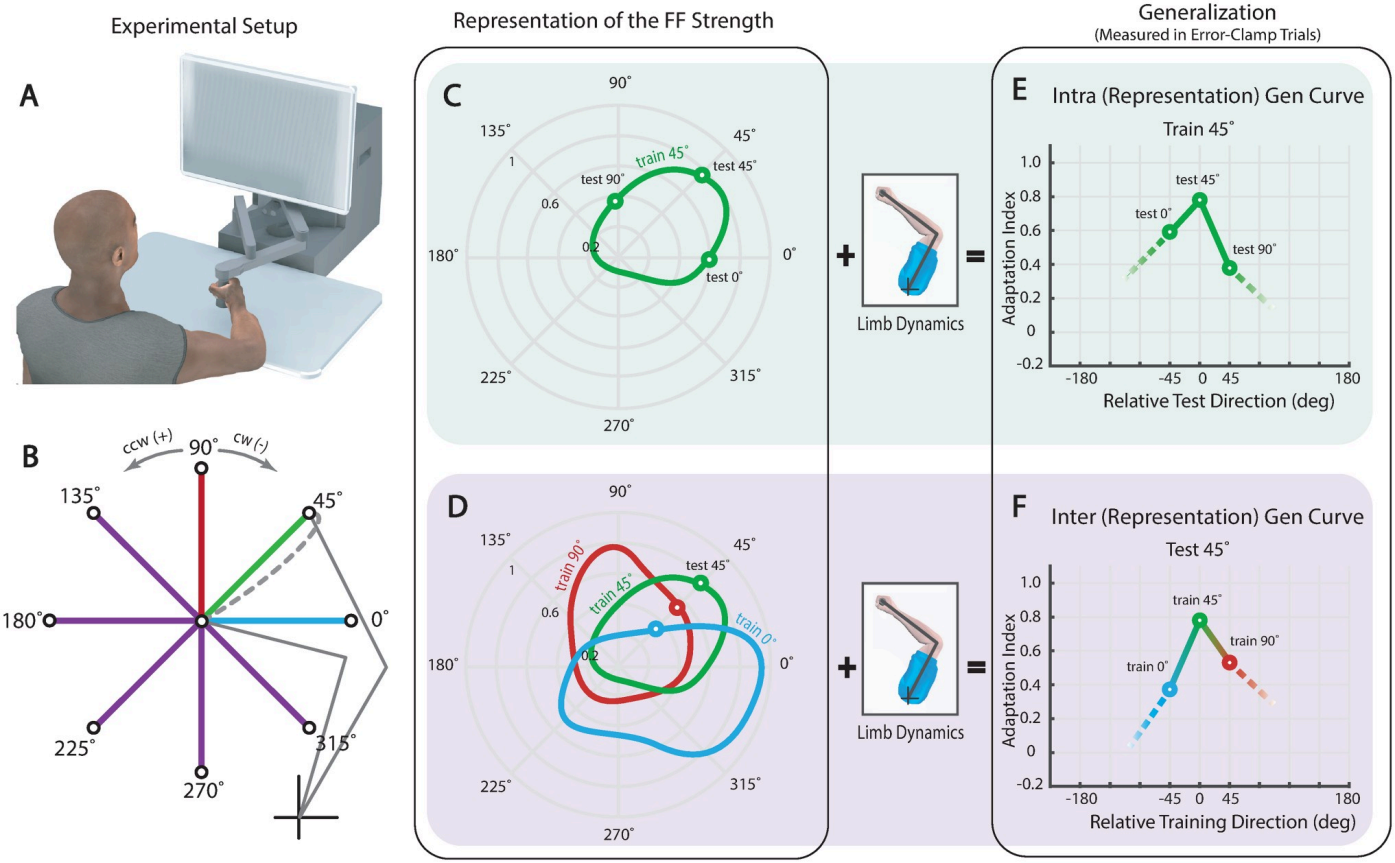
Experimental setup and generalization curves. (**A**) Subjects made reaches in a null field, a curl field or an error-clamp. After landing on target the robot brought their hand back to the center position. (**B**) Eight standard targets were located 10-cm away from a home target. Each of eight groups practiced reaching in a CW force field towards one of the eight targets (the gray dashed line depicts a reach in the field to the 45° target) and then tested for generalization to all remaining targets using error-clamp trials. (**C**) A schematic of the internal representation of the learned force field for the 45° target group. The curve depicts a subject’s estimated strength of the force field in polar coordinates (green contour). (**D**) Using this estimate the limb makes a generalization reach. Combining the measurements made from many such reaches after training on the 45° target constitutes an intra-representation generalization curve (green circles). These measurements are influenced by both the force field estimate and changes in limb impedance with reach direction. (**E**) Schematic of representations from three groups of subjects learning the field for the 0° (blue contour), 45° (green contour), and 90° (red contour) targets. (**F**) Combining the measurements made across all three groups when reaching to the same 45° target constitutes an intra-representation generalization curve. This curve corrects for the changes in reach direction.

**Fig 2:**
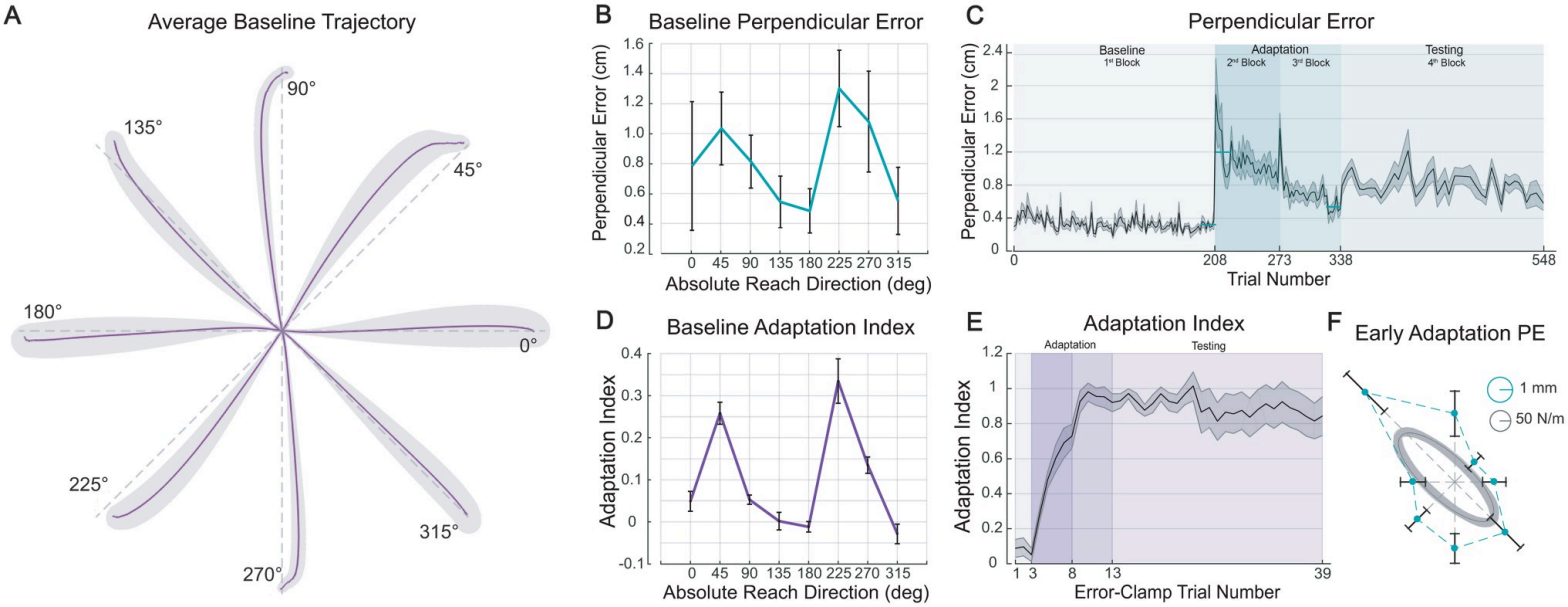
Experiment’s behavioral results. (**A**) Baseline reaches in null field made with visual feedback (across-subject averages ± SEM). Reaches slightly deviate from a straight path (gray dashed lines). (**B**) Baseline perpendicular errors averaged across subjects. (**C**) Across-subject average perpendicular errors (±SEM) are plotted across all trials of the experiment. Only error-clamp trials are excluded. Subjects practiced reaching in force field during the 2^nd^ and 3^rd^ blocks with a brief rest between. (**D**) Baseline adaptation index averaged across subjects. Note the qualitatively similar shape to the perpendicular errors in **B**. (**E**) Force field learning curve averaged across all subjects and all reach directions. Mean values to the training targets (±SEM) are plotted across error-clamp trials. (**F**) Early adaptation perpendicular errors (mean ±SEM of first 20 trials) are averaged across subjects and overlaid on a hand stiffness ellipse from [7]. Note the qualitatively similar shapes of errors and hand stiffness.

## References

[1] Rezazadeh A, Berniker M (2019) Force field generalization and the internal representation of motor learning. PLoS ONE 14(11): e0225002 10.1371/journal.pone.0225002 31743347 PMC6863527

