# Supplementary figures and images for: Force field generalization and the internal representation of motor learning
Source: PLoS One. 2019 Nov 19;14(11):e0225002. doi: 10.1371/journal.pone.0225002 (PMC6863527; doi:10.1371/journal.pone.0225002)

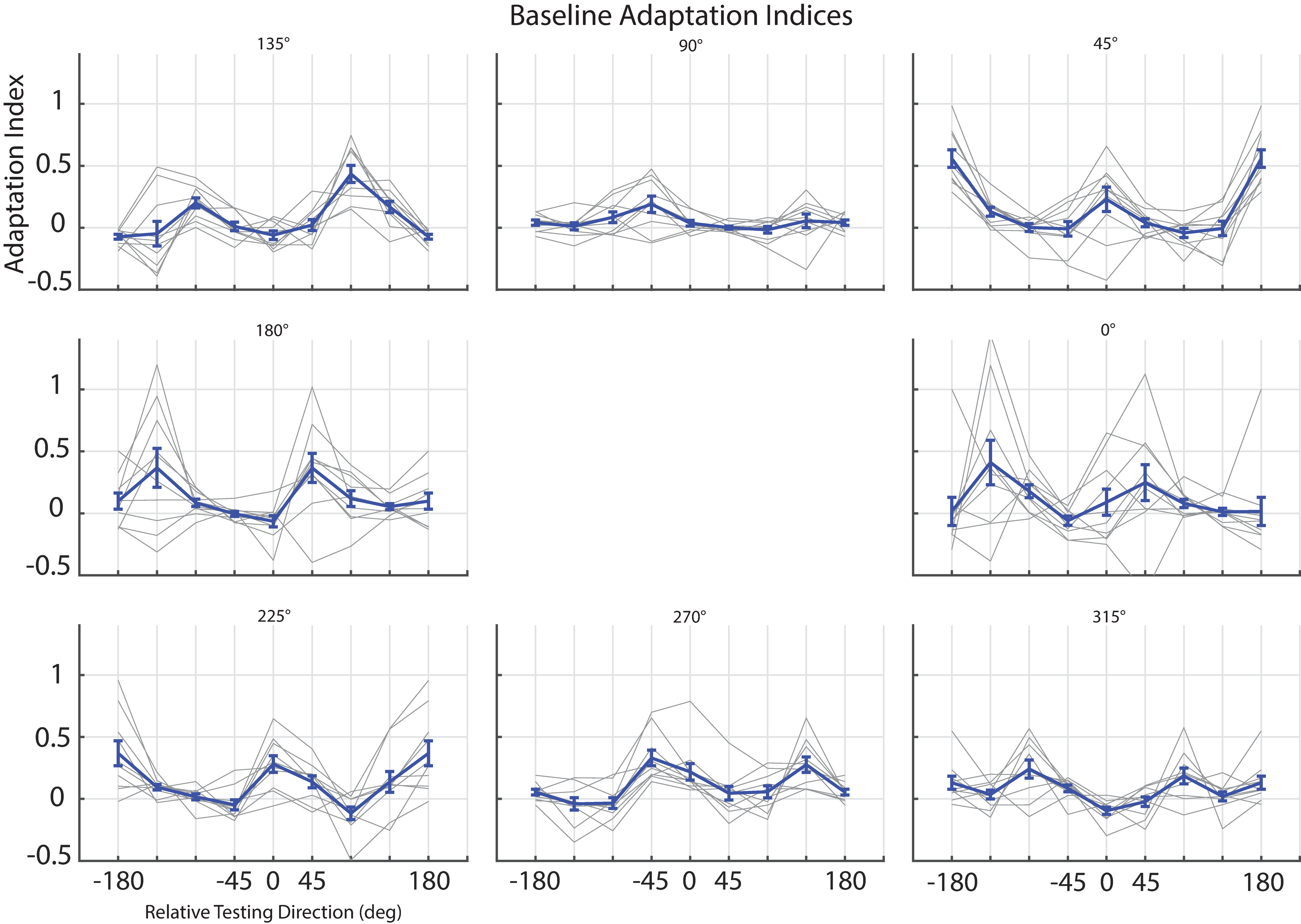

Supplement: S1 Fig — Average adaptation indices (gray) measured in baseline channel trials towards the eight target directions for individual subjects in each of eight groups (10 subjects per group). Across-subject’s average for each group is also displayed (averages ± SEM, blue). Directions on top of each panel refers to the direction of the, yet to be presented, force field for each group later in adaptation trials. (TIF) [file pone.0225002.s001.tif]

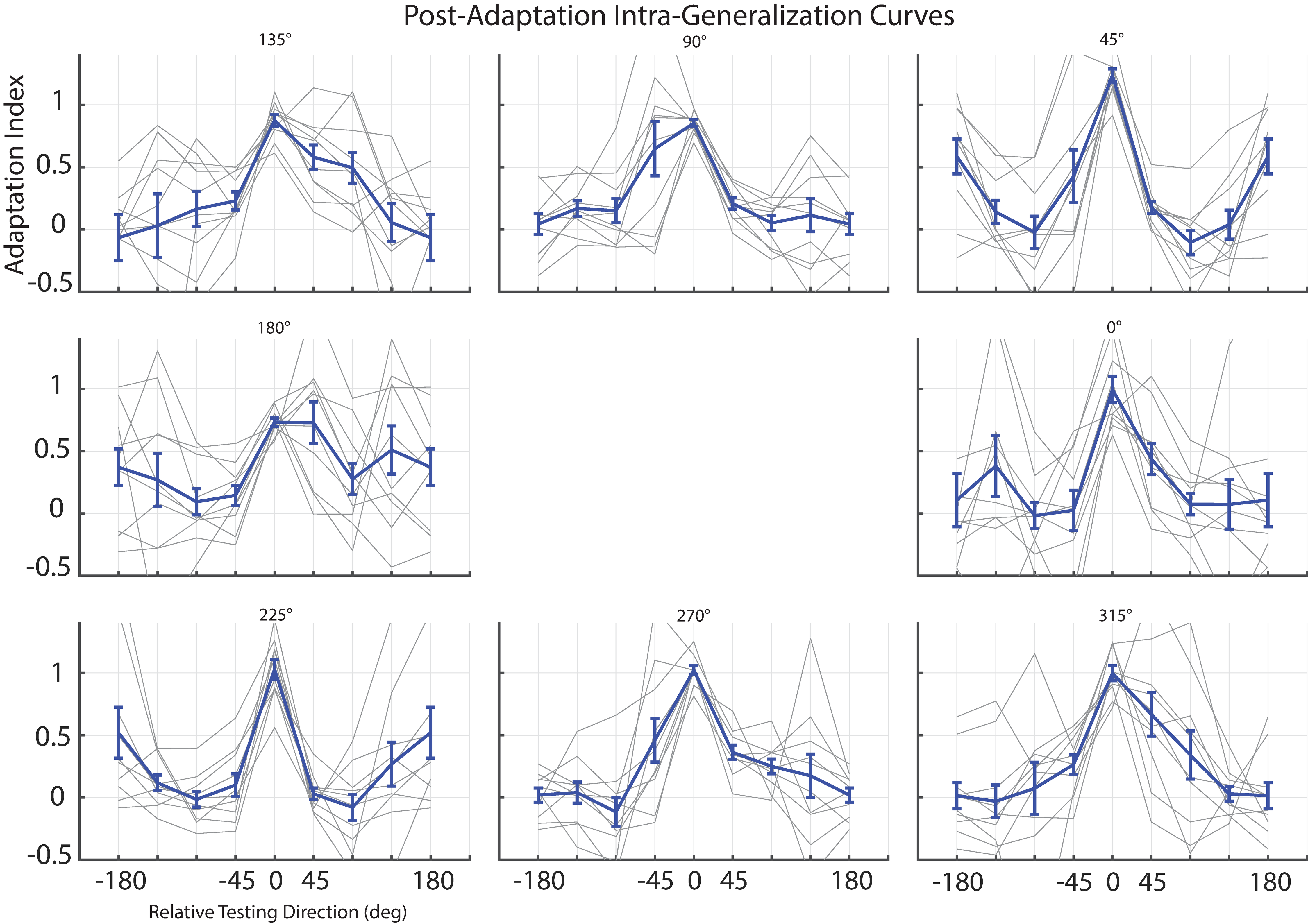

Supplement: S2 Fig — Average adaptation indices (gray) measured in test block channel trials towards the eight target directions for individual subjects in each of eight groups (10 subjects per group). Across-subject’s average for each group is also displayed (averages ± SEM, blue). Directions on top of each panel refers to the direction of the force field for each group in adaptation trials. (TIF) [file pone.0225002.s002.tif]

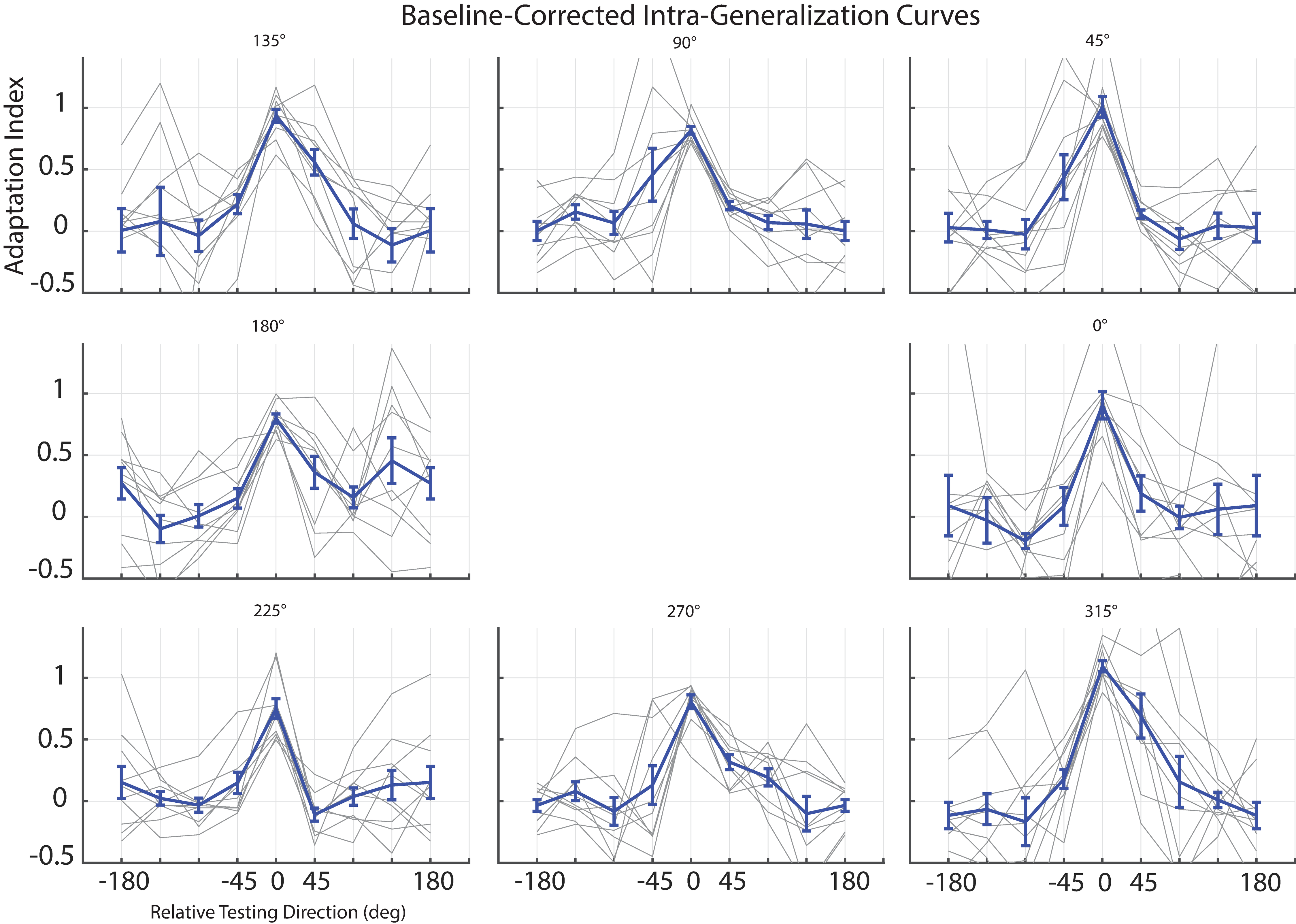

Supplement: S3 Fig — Average adaptation indices (gray) measured in test block channel trials towards the eight target directions for individual subjects in each of eight groups (10 subjects per group) corrected for the baseline indices (See Fig A-1). Across-subject’s average for each group is also displayed (averages ± SEM, blue). Directions on top of each panel refers to the direction of the force field for each group in adaptation trials. (TIF) [file pone.0225002.s003.tif]
